# From yeast to hypha: defining transcriptomic signatures of the morphological switch in the dimorphic fungal pathogen *Ophiostoma novo-ulmi*

**DOI:** 10.1186/s12864-016-3251-8

**Published:** 2016-11-15

**Authors:** M. Nigg, L. Bernier

**Affiliations:** 1 Institut de Biologie Intégrative et des Systèmes (IBIS), Université Laval, Room 2255, Pavillon Charles-Eugène-Marchand, 1030, Avenue de la Médecine, Québec, Québec G1V 0A6 Canada; 2 Département des sciences du bois et de la forêt, Centre d’Étude de la Forêt (CEF), Université Laval, Québec, G1V 0A6 Canada

**Keywords:** Yeast-to-hypha transition, RNAseq, Time-course experiment, *Ophiostoma novo-ulmi*, MAPK cascade

## Abstract

**Background:**

Yeast-to-hypha transition is a major morphological change in fungi. Molecular regulators and pathways that are involved in this process have been extensively studied in model species, including *Saccharomyces cerevisiae*. The Mitogen-Actived Protein Kinase (MAPK) cascade, for example, is known to be involved in the yeast-to-pseudohypha switch. Yet the conservation of mechanisms regulating such morphological changes in non-model fungi is still poorly understood. Here, we investigate cell remodeling and transcriptomic modifications that occur during this morphological switch in the highly aggressive ascomycete fungus *Ophiostoma novo-ulmi*, the causal agent of Dutch elm disease.

**Results:**

Using a combination of light microscopy, scanning electron microscopy and flow cytometry, we demonstrate that the morphological switch occurs in less than 27 h, with phenotypic cell modifications being detected within the first 4 h. Using RNAseq, we found that over 22% of the genome of *O. novo-ulmi* is differentially expressed during the transition. By performing clustering analyses of time series gene expression data, we identified several sets of genes that are differentially expressed according to distinct and representative temporal profiles. Further, we found that several genes that are homologous to *S. cerevisiae* MAPK genes are regulated during the yeast-to-hypha transition in *O. novo-ulmi* and mostly over-expressed, suggesting convergence in gene expression regulation.

**Conclusions:**

Our results are the first report of a time-course experiment monitoring the morphological transition in a non-model Sordariomycota species and reveal many genes of interest for further functional investigations of fungal dimorphism.

**Electronic supplementary material:**

The online version of this article (doi:10.1186/s12864-016-3251-8) contains supplementary material, which is available to authorized users.

## Background

*Ophiostoma novo-ulmi* (Ascomycota, Sordariomycetes) is the highly aggressive dimorphic pathogen that is responsible for the ongoing pandemic of Dutch elm disease (DED) [1]. This fungus is capable of assuming two distinct forms: a unicellular yeast stage and a multicellular hyphal form. In the context of DED, both forms are thought to be involved in pathogenesis. The yeast cells are considered to be responsible for the passive colonization of the xylem vessels as they are carried by the sap flow and found rapidly after invasion both in the shoots and in the roots [2]. The hyphal form (also known as the invasive phase) is suggested as being responsible for fungal dispersion between adjacent vessels by passing through the vessel pits. This pluricellular form is also very important during the saprophytic stage of *O. novo-ulmi* for the colonization of the principal egg galleries that are dug by female elm bark beetles on dead trees and further into secondary galleries that are excavated by the beetle larvae [3].

Many species within the genus *Ophiostoma* are known to be dimorphic, including the other two DED fungi, *O. ulmi* and *O. himal-ulmi* as well as the sapwood-staining fungi *O. piceae* [4, 5] and *O. quercus* [6]. Few studies have been devoted to the identification of key factors that regulate the yeast-to-hypha (Y-to-H) transition within this genus. Nutritional factors, such as nitrogen sources [7, 8], pyridoxine [9] or linoleic acid [10], have been shown to be involved, together with other molecular factors, such as Ca^2+^-calmodulin interaction [11, 12] or cyclic Adenosine MonoPhosphate (cAMP) [13]. Inoculum size is also implicated in the regulation of the Y-to-H transition in DED fungi and effects have been confirmed for *O. ulmi*, both subspecies of *O. novo-ulmi* (*americana* and *novo-ulmi*), and *O. himal-ulmi* [8, 14–16]. Moreover, quorum sensing has been shown in *O. ulmi, O. piceae and O. floccosum* [15, 17, 18]. Until now, only two genes have been identified as being involved in the dimorphism in *O. novo-ulmi*, namely *COL1* [19] and an as yet uncharacterized gene that also affects asexual sporulation (synnematospores) and pathogenicity [20, 21]. The knockout *col1* mutant exhibits reduced mycelial growth, whereas yeast growth is barely affected by the mutation [19]. However, pathways and genes regulating the Y-to-H transition in *O. novo-ulmi* are still largely unknown.

Dimorphism in fungi is a morphological characteristic that has been studied for many years in model species, such as *Saccharomyces cerevisiae*, the human pathogens *Candida albicans* and *Histoplasma capsulatum,* and the plant pathogen *Ustilago maydis*. The hyphal state is very variable among species, and is represented by pseudo-hyphae in *S. cerevisiae* (not common in nature) or by true septate hyphae in filamentous fungi. In all of these species, several conserved pathways have been reported to be linked to the Y-to-H switch, including the Mitogen-Activated Protein Kinase (MAPK) cascade [22–24], the Protein Kinase A (PKA) pathway [25–27], and the pH-dependent RIM pathway [28, 29].

**Fig. 1:**
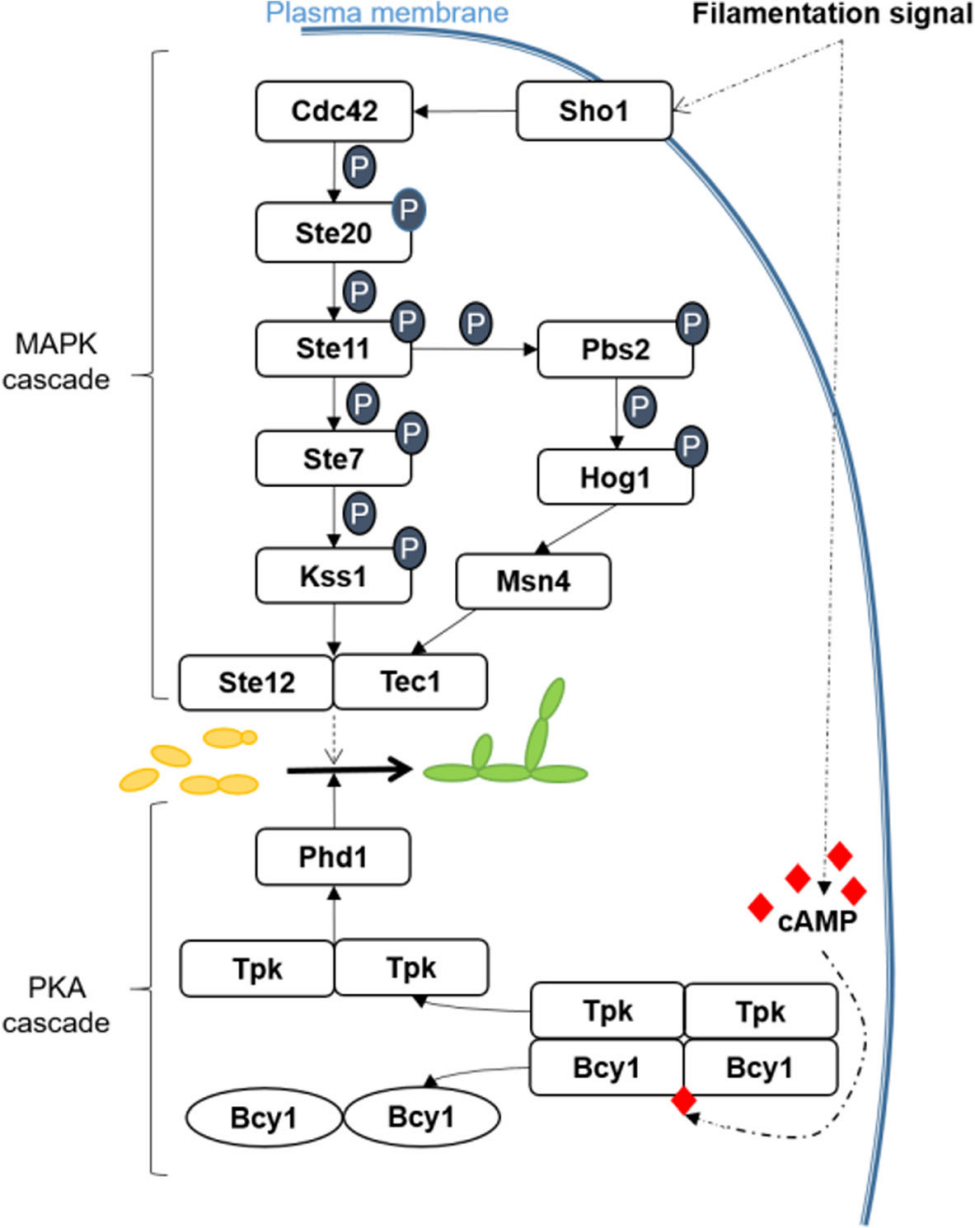
Simplified and non-exhaustive illustration of the *S. cerevisiae* proteins involved in the MAPK and cAMP-PKA cascades that regulate pseudohyphae formation. *Dashed arrows* indicate indirect regulation through other components that are not displayed here. P: phosphate (PO^3−^)

In *S. cerevisiae*, the MAPK cascade that is involved in morphological changes is well described (for a simplified schematic, see Fig. 1). The initial signal is perceived by the osmoreceptors Sho1 and Msb2, which are located on the plasma membrane. The signal is then transduced to a first kinase Ste20, and a cascade of phosphorylations is activated through Ste11 (MAPKKK), Ste7 (MAPKK), and Kss1 (MAPK). The unphosphorylated Kss1 acts as a repressor of the morphological switch since it sequestrates the transcription factors Tec1 and Ste12 in the nucleus. The phosphorylation of Kss1 by Ste7 induces the release of Tec1 and Ste12 [23, 30, 31]. An alternative pathway involves the activation by Ste11 of another MAPKK, Pbs2, and then by Hog1, a MAPK that responds to hyperosmolar conditions [32, 33]. Hog1 activates Msn4, which is a stress-response transcriptional factor that is linked to Ste12 [33]. In *C. albicans*, both cascades (via Ste7 or Hog1) are conserved and involved in the control of morphogenesis [34, 35].

The PKA protein of *S. cerevisiae* is a tetramer composed of a homodimer of regulator sub-units (Bcy1) and a homo- or heterodimer of catalytic sub-units (two of the three possible Tpk1, Tpk2 or Tpk3 sub-units). The activation of the PKA pathway depends upon the presence of cAMP [36] (for a simplified schematic, see Fig. 1). Of the three Tpk proteins, only Tpk2 exerts a positive effect on pseudohyphal growth in *S. cerevisiae*, since deletion of *Tpk2* represses filamentous growth. In contrast, *Tpk1* deletion has no effect, while knockout mutants of *Tpk3* exhibit hyperfilamentous growth [36, 37]. Both Tpk1 and Tpk2 regulate genes that are involved in the formation of pseudohyphae. Tpk1 regulates the activity of the dual-specificity tyrosine-regulated kinase Yak1, which controls the expression of *Flo11. Flo11* is a gene encoding a cell surface flocculin through the transcription factors Sok1 and Phd1 [38]. Through phosphorylation, Tpk2 activates the transcriptional factor Flo8 which, in turn, activates the expression of filamentation target genes, such as *Flo11* [36]. Also through Tpk2, the PKA pathway induces the activation of the transcriptional factor Phd1. PKA is highly conserved in *C. albicans* and the Phd1 *S. cerevisiae* homolog, Efg1, is also activated through Tpk2 [27]. Both Phd1 and Efg1 are important regulators of dimorphism [22, 39].

Finally, the RIM pathway, which is also called the PAL cascade in filamentous fungi, is dependent on variation in pH within the environment. In *C. albicans* and *S. cerevisiae* a change from acidic to neutral pH induces the Y-to-H switch (Y-to-pseudohyphae in *S. cerevisiae*) [40, 41], whereas hyphal form in *U. maydis* is favoured by acidic pH [41]. The PAL/RIM pathway involves at least six proteins which regulate the activation of a zinc-finger-like transcriptional factor called PacC or RIM101 [28]. RIM101 regulates, in turn, the expression of downstream genes, such as *PHR1* and *PHR2* in *C. albicans* (*GAS1* in *S. cerevisiae*) [41].

A few large-scale transcriptomic analyses have been conducted to determine the molecular regulation of the Y-to-H transition in model species. RNAseq was recently used in *Penicillium marneffei* where 2718 genes (28% of the gene content) were differentially expressed during the morphological change [42]. DNA microarrays in *Candida albicans* were used under different conditions to identify sets of genes that are regulated during the transition [43–45]. Yet genome-wide analyses of this major morphological change, which would describe the global cell response to Y-to-H transition-promoting stimuli, are still lacking. Indeed, most of the studies focus on a particular protein or pathway.

The *O. novo-ulmi* genome was recently sequenced [46] and almost 75% of the 8640 predicted genes have since been annotated [47], thereby facilitating further genomic studies. Comparative analyses of the transcriptomes of yeast and mycelial forms of *O. novo-ulmi* strain H327 [48] show a clear difference in gene expression profiles between the two life stages and reveal processes that are specific to each form. Based on this study, we investigate the regulation of gene expression during the Y-to-H transition using a time-series approach, which allows the description of dynamic biological processes. We used RNAseq technology to characterize major molecular changes that are associated with the morphological switch. We also focused attention on the regulation of gene expression in *O. novo-ulmi* MAPK, PKA and RIM pathway homologs.

## Results

### Microscopy and flow cytometry

**Fig. 2:**
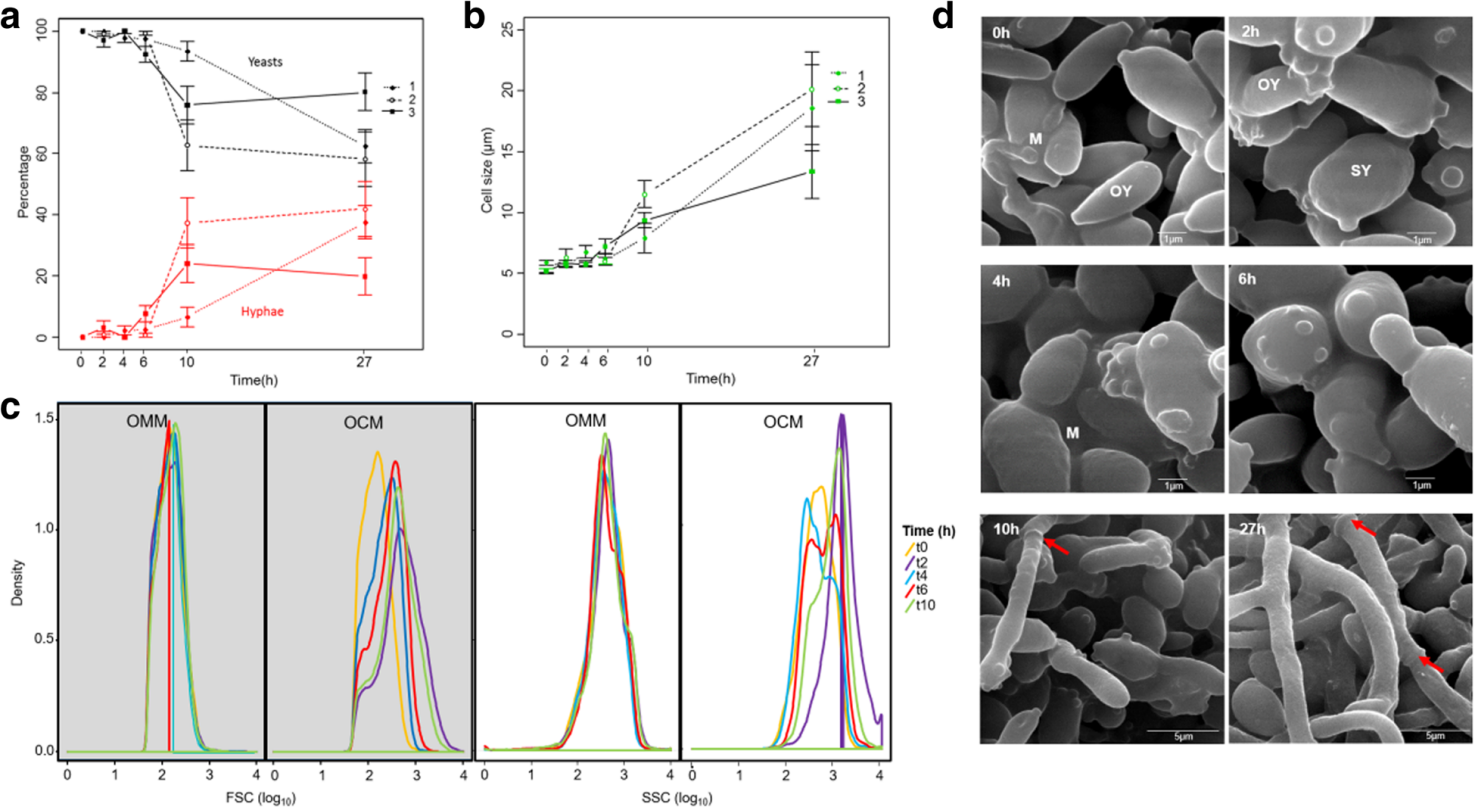
**a** Percentage of yeast and mycelium in *Ophiostoma novo-ulmi* at 0, 2, 4, 6, 10 and 27 h of incubation in conditions inducing the yeast-to-hypha transition. This was achieved by transferring yeast cells grown in shake liquid minimal medium to fresh liquid complete growth medium (OCM) without agitation. Values are means and standard deviations for three replicates. *Black*: yeast percentage; *Red*: Hyphae percentage. **b** Cell size in μm at each of the 6 time points for the three replicates. **c** Density of cell size (*gray panels*) and density of cell granularity or internal complexity (*white panels*) at each of 5 time points measured by flow cytometry. FSC: Forward Scatter, cell size. FSC: Side Scatter, granularity. OMM: minimum medium under agitation, no transition. OCM: *Ophiostoma* complete medium in still conditions, switch from yeast to mycelium. **d** Scanning-electron microscopy pictures taken at each time point. Scale is indicated on the pictures. OY: ovoid yeast cells; SY: spherical yeast cells; M: extracellular matrix. *Red arrows* indicate septa

We first defined the Y-to-H transition in *O. novo-ulmi* by recording the modifications that were observed at the population and cell levels using light microscopy. During the Y-to-H transition that was induced by incubation in still-liquid culture in OCM (complemented with proline), the percentage of yeast cells decreases within the first 10 h following transfer to the induction medium, while the percentage of mycelium increases (Fig. 2a). Further, cell size increases with time (Fig. 2b).

The Y-to-H transition was then followed using flow cytometry. We compared conditions that do not favor transition (shake-liquid OMM complemented with proline [7, 8], Fig. 2c) with the condition that was selected in this study (Fig. 2c). We observe that both cell size (FSC) and cell internal granularity (SSC) vary through time under conditions that induce the Y-to-H transition. Within the first 10 h, peaks of density shift towards higher FSC and SSC. Finally, scanning electron micrographs (SEM) that were taken at each time point confirm the Y-to-H transition and highlight cell shape modification within the first 4 h, as cells may switch from an ovoid (OY, Fig. 2d) to a spherical shape (SY).

### Global view of transcriptomes of yeast-to-hypha transition

For each of the six time points that were previously defined by microscopy and flow cytometry (i.e., 0, 2, 4, 6, 10 and 27 h after transfer to the induction medium), samples were collected and total RNA was extracted to sequence the whole transcriptome. After filtering and trimming, RNAseq samples contained between 7 and 12 million reads (Table 1). By alignment on the complete set of exons of the *O. novo-ulmi* genome, we are able to achieve from 25.4 up to 63.6 X exon coverage depth.

**Table 1:** General characteristics of the 18 RNAseq samples after trimming and filtration

Samples	Replicates	Time (h)	No. of reads^a^	No. of reads mapped	No. of bases	Exon coverage depth	No. of genes expressed^b^
MN1	1	0	11968893	6550928	790686427	54.0X	7413
MN2	1	2	12899265	7054621	881079467	60.2X	7436
MN3	1	4	12739833	7197167	897760976	61.3X	7465
MN4	1	6	13641971	7461971	930946766	63.6X	7515
MN5	1	10	8949640	4863675	604538988	41.3X	7487
MN6	1	27	9109003	4840961	558218816	38.1X	7543
MN7	2	0	7382503	3826743	454704414	31.0X	7256
MN8	2	2	9425687	4860220	594473189	40.6X	7420
MN9	2	4	9259734	5094283	630655472	43.1X	7418
MN10	2	6	9418078	5124364	614559004	42.0X	7468
MN11	2	10	11072018	6185630	751150263	51.3X	7551
MN12	2	27	7301344	3167396	371782642	25.4X	7531
MN13	3	0	11782413	6722999	812905343	55.5X	7401
MN14	3	2	8145563	4536653	567033310	38.7X	7306
MN15	3	4	11419771	6636608	827274989	56.5X	7408
MN16	3	6	10960584	6445652	797790606	54.5X	7550
MN17	3	10	12468947	7297888	900860683	61.5X	7570
MN18	3	27	7876023	4271786	504251284	34.4X	7580

*No.* number

^a^All reads are 25–150 nt long

^b^At least one read after normalization in EdgeR

**Fig. 3:**
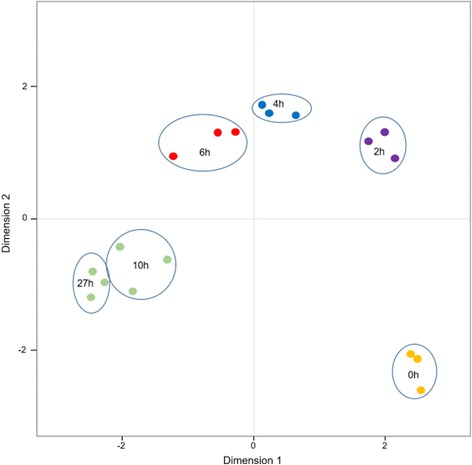
Multidimensional Scaling (MDS) plot of the 18 RNA samples sequenced during the yeast-to-hypha transition in *Ophiostoma novo-ulmi. Dot colors*: results of cluster analysis by *k*-means test. *Dots* from the same color represent values that are not significantly different according to the *k*-means test. *Blue circle*: contains the three replicates of each time point (0, 2, 4, 6, 10 and 27 h after transfer to fresh complete medium)

We retained a total of 7605 genes expressed under our experimental conditions out of the 8640 genes that are predicted in *O. novo-ulmi* for downstream analyses. Each sample contains at least 7256 genes that are represented by more than one read (Table 1, Additional file 1: Figure S2, Additional file 2: Table S3). Multidimensional scaling (MDS) analysis shows that the samples cluster according to the time after transfer to OCM and, within-time points, the three biological replicates cluster together (Fig. 3). The length of incubation of cells in non-agitated OCM (0–27 h) explains most of the variability in the data (first axis). Molecular variability is small between 10 and 27 h. In the second dimension, there is a large difference between samples that were collected at the beginning of the experiment and those that were collected as early as 2 h after transfer to non-agitated OCM.

### Identification and analysis of differentially Expressed Genes (DEGs)

**Fig. 4:**
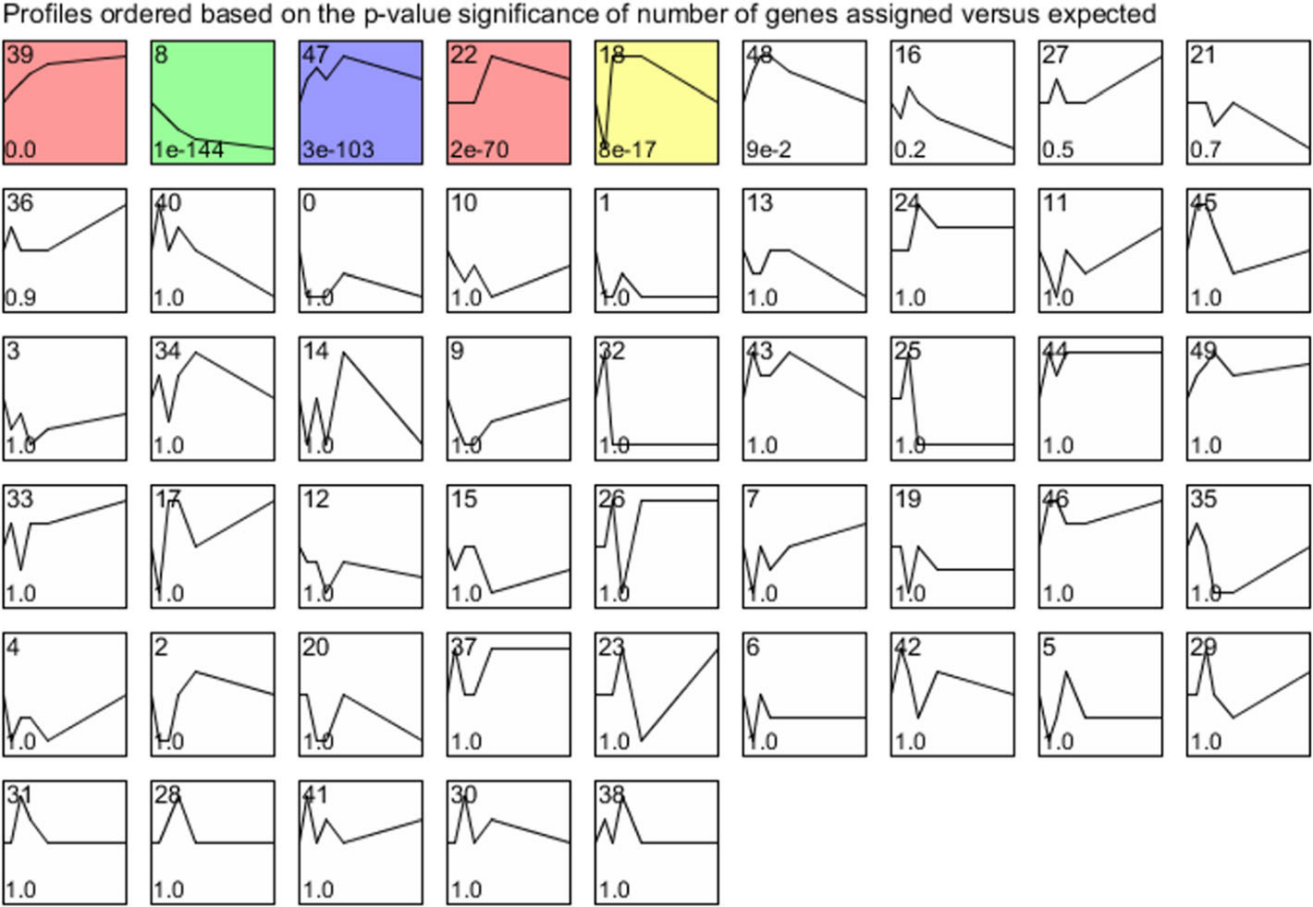
Expression profiles of genes during yeast-to-hypha transition in *Ophiostoma novo-ulmi* assessed using STEM on differentially expressed genes identified using *Next-maSigPro* package. Profiles are ordered based on the *P*-value significance of number of genes assigned versus expected*. Numbers on the top right*: profile ID; numbers on the bottom right: *P*-value significance of number of genes assigned versus expected. *Colored profiles*: *P*-value significant (corrected with FDR). *Profiles* with the same color are part of a unique cluster

**Fig. 5:**
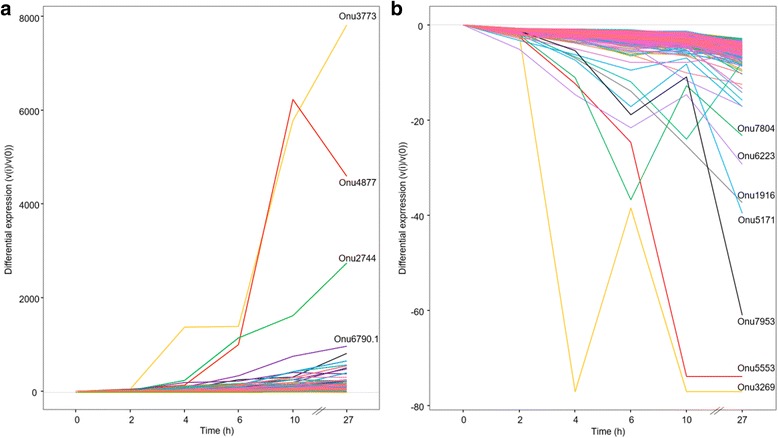
Differential gene expression along time points for genes in STEM profiles 39 (**a**) and 8 (**b**). Top genes for each profile are highlighted

The *Next-maSigPro* package in R (v3.0.1) applies generalized linear models (GLM) to regression models in order to determine differentially expressed genes (DEGs) in RNAseq time-course analyses [49, 50]. Here, when the package was used on normalized read counts, a total of 1897 genes out of the 7605 expressed genes were identified as DEGs during the Y-to-H transition (0–27 h), when a false rate discovery (FDR) threshold [51] of 0.05 was employed. (Additional file 2: Table S3). This set of DEGs was then analyzed by clustering methods with STEM software [52, 53]. Here, we present results for the assignment of DEGs to 50 predefined gene expression model profiles (0–49) (Fig. 4). A total of five profiles are identified as containing more genes than expected by chance (significant *P*-values that were corrected for FDR, ranging from 72 to 815 genes, colored profiles). Some genes are present in more than one profile (Additional file 2: Table S4). The five profiles were then consolidated into four clusters that gather together similar model profiles, which were represented by different colors, with one cluster including two profiles (red, Fig. 4). We performed a more in depth investigation of profiles 39 (815 genes, 9.4% of the total gene content) and 8 (256 genes, 3% of the total gene content), since they are the most significant (*P*-values of 0 and 1e^−144^, respectively) and exact opposites in terms of gene expression regulation (Figs. 4 and 5a, b).

#### Genes with increasing expression during Y-to-H transition (profile 39)

Profile 39 comprises genes that are increasingly expressed during the Y-to-H transition. The most notable gene in this profile is Onu3773, which is annotated as coding for an extracellular serine-threonine-rich protein, and also related to the Adhesin protein Mad1 (Additional file 2: Table S4). This gene is over-expressed by 7819 times at 27 h compared to 0 h. Another notable gene is Onu6790.1, which is annotated as coding for the Woronin body major protein, and is homologous to Hex1 in *O. floccosum*. In *O. novo-ulmi*, this gene is barely expressed at the beginning of the switch, but its expression increases rapidly within the 27 first hours (959 times more strongly expressed at 27 h than at 0 h; Additional file 2: Table S4). Genes coding for actin, tubulin and septin are also present in profile 39. Of the 67 genes encoding carbohydrate-active enzymes (CAZymes) that were found within the total set of DEGs, 31 genes are grouped in this profile. This set includes glycosyltransferases (GT, *n* = 21), glycoside hydroxylases (GH, *n* = 8) and carbohydrate binding modules (CBM, *n* = 2) (Additional file 2: Table S4). Finally, 90 genes found in profile 39 have been already described as being over-expressed in the mycelium growth phase of *O. novo-ulmi* [48] (Additional file 2: Table S3). By gene ontology (GO) term-enrichment analysis, we determined that 112 biological processes (BPs) containing more than four genes are significantly enriched (*P*-value ≤ 0.01) in profile 39 (Additional file 2: Table S5). After using REVIGO software [54] to reduce redundancy, 28 terms were retained (Additional file 3: Figure S3). Among the enriched BPs, many are related to cellular organization, including localization (GO:0051179, 127 genes), cellular component organization (GO:0016043, 67 genes) and microtubule-based movement (GO:0007018, 10 genes) (Additional file 2: Table S5). Cell division, vesicle transport and protein modifications are also dominant processes.

#### Genes with decreasing expression during Y-to-H transition (profile 8)

Profile 8 comprises genes for which expression decreases during the Y-to-H transition (Additional file 2: Table S6). Only four genes encoding CAZymes (three GHs and one GT) are found. Gene Onu5171 is one of the top genes in the profile (40 times more repressed at 27 h compared to 0 h), and encoding a siderophore iron transporter 1 (Ferrioxamine B permease). A total of 15 genes that were associated with yeast growth conditions by Nigg et al. [48] are down-regulated during the Y-to-H transition and are present in profile 8 (Additional file 2: Table S3). In this profile, we further found gene Onu8210, which was annotated as encoding a DNA-binding protein. This protein has a zinc-finger DNA binding domain, which is homologous to the domain of *S. cerevisiae* Mig1, Nrg1 and Msn4 proteins (data not shown). Gene Onu8210 is down-regulated during the Y-to-H transition (5.75 times less expressed at 27 h after transfer to OCM).

Finally, GO term enrichment analysis that was followed by the application of REVIGO highlighted 22 BPs and revealed over-representation of terms that were associated with non-coding RNA (ncRNA) metabolism and ribosome biogenesis (Additional file 4: Figure S4 and Additional file 2: Table S7).

#### Other genes of interest

Gene Onu6217-*CAT1* homolog, which has already been described as being over-expressed in the yeast form compared to the mycelium phase [48], is highly down-regulated along the Y-to-H transition (profile 0, 192 times less expressed after 2 h) (Additional file 2: Table S3). Another gene, Onu7070, which codes for a type 1 endochitinase, was previously reported as being over-expressed in the mycelium phase [48]. This gene is also regulated during the Y-to-H switch and ends up being 14 times more strongly expressed at 27 h compared to 0 h (profile 27) (Additional file 2: Table S3). Gene Onu4296 codes for Cerato-ulmin (CU), a hydrophobin that has been well studied in the context of DED. This gene is down-regulated through time and belongs to profiles 0 and 1 (Additional file 2: Table S3).

### Identification and expression analysis of *S. cerevisiae* MAPK, PKA, and RIM pathway homologs

#### *S. cerevisiae* MAPK protein homologs

**Fig. 6:**
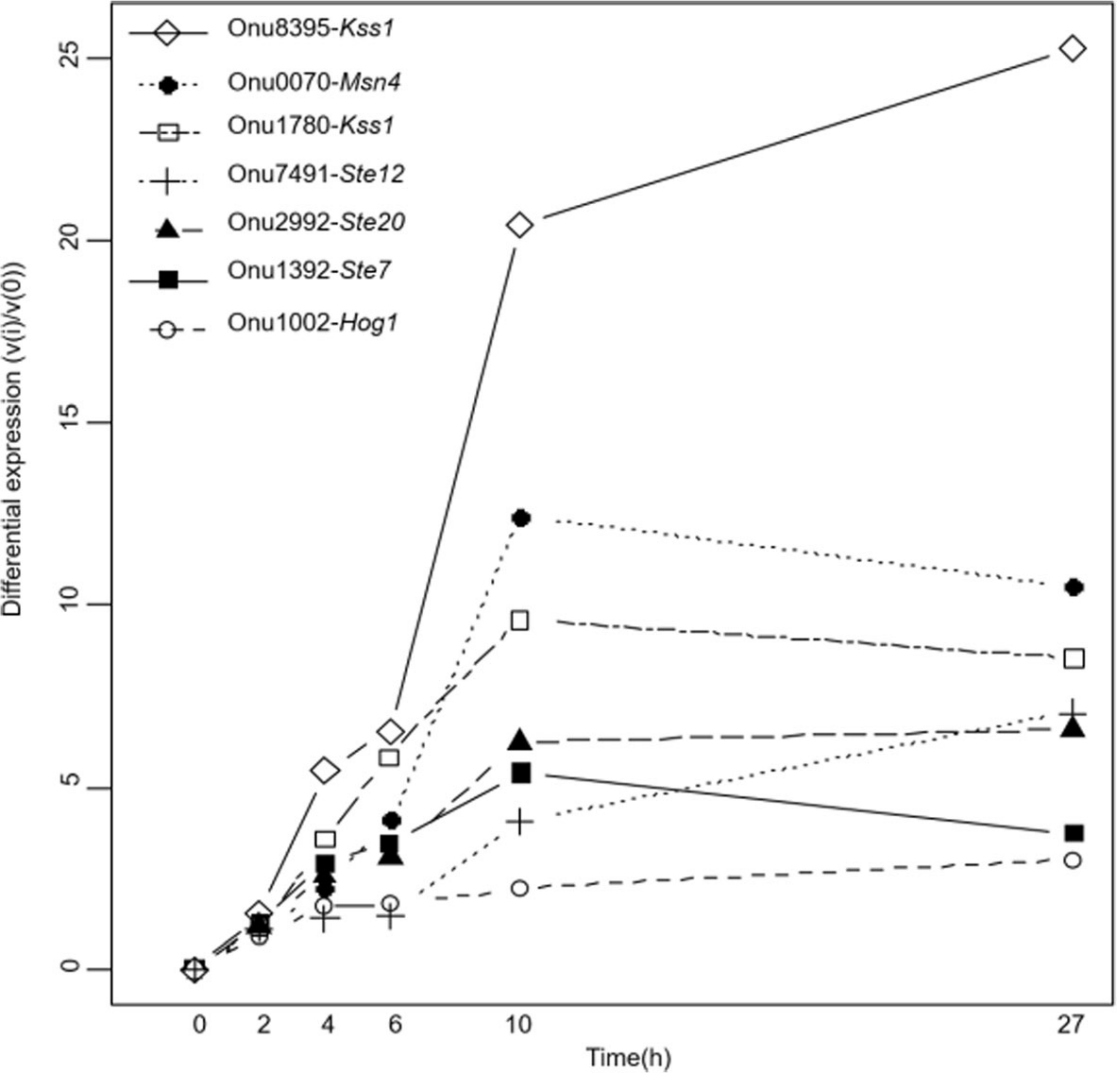
Differential gene expression for *Ophiostoma novo-ulmi* homologs to genes implicated in the MAPK cascade in *S. cerevisiae*. Differential gene expression is the difference in read count between the point of interest (i) and the start point of the experiment (0), calculated by STEM software [52, 53]

We identified genes coding for potential proteins that were homologous to *S. cerevisiae* MAPK proteins. Of the 11 *S. cerevisiae* proteins with homologs in *O. novo-ulmi*, four have more than one potential homolog (Ste20, Kss1, Sho1 and Msn4; Additional file 2: Table S2). Eight genes encoding these homologous proteins are actually orthologs: Onu0581-*Ste20*, Onu2999-*Ste11*, Onu4018-*Pbs2*, Onu1002-*Hog1*, Onu2279-*Cdc42*, Onu7491-*Ste12*, Onu1392-*Ste7*, and Onu5199-*Tec1* (Additional file 2: Table S2). We found the following eight genes to be differentially expressed during the Y-to-H transition: Onu1002-*Hog1*, Onu2992-*Ste20*, Onu1392-*Ste7*, Onu7491-*Ste12*, Onu0070-*Msn4*, Onu8210-*Msn4*, Onu1780-*Kss1* and Onu8395-*Kss1* (Fig. 6b). *Hog1*, *Ste20*, *Ste7*, *Kss1* and *Ste12* homologs are all over-expressed during the Y-to-H transition and are present in STEM profile 39 (Fig. 6b).

#### *S. cerevisiae* cAMP-PKA protein homologs

We found homologous genes for the most important genes that were involved in the cAMP-PKA pathway of *S. cerevisiae* (*Bcy1*, *Tk1*-*3*, *Yak1*, *Phd1, Sok2, Flo8* or *Pde2*; Additional file 2: Table S2). Only Onu6515-*Bcy1* and Onu7499-*Pde2* are orthologous genes. Phd1 and Sok2 are putative homologs of the same protein in *O. novo-ulmi*, Onu0977. Surprisingly, we did not find a homologous protein for Flo11. The gene encoding the best homologous match for *Yak1*, i.e.*,* Onu0836, is the only one that is differentially expressed under our set of experimental conditions. This gene is part of profile 39 and is already over-expressed almost 4-fold at 4 h following induction of the transition. For genes Onu0977-*Phd1/Sok2* and Onu7708-*Tpk,* large differences among biological replicates might explain why these genes do not pass the threshold set for DEG identification.

#### *S. cerevisiae* RIM protein homologs

All six well-described proteins that are involved in the RIM pathway have a homolog in *O. novo-ulmi* (Additional file 2: Table S2) and five of them are actually encoded by orthologous genes. However, Onu0751-*RIM101* is the only gene that is differentially expressed during the Y-to-H transition. Here, this gene is down-regulated (12-fold lower at 27 h compared to 0 h) and is present in profile 0 (Additional file 2: Table S3).

### Quantitative Reverse Transcription-PCR (qRT-PCR) analysis of selected genes

In order to confirm RNAseq data, we chose genes that were detected as being DE and tested their expression using qRT-PCR. We selected genes from the MAPK pathway (Onu1392-*Ste7*, Onu7491-*Ste12*, Onu8395-*Kss1,* Onu1002-*Hog1* and Onu8210-*Msn4*) and genes with contrasting expression profiles: over-expressed genes (Onu0980 and Onu6970.1-*Hex1*) and down-regulated genes (Onu0303 and Onu5171). For all nine genes, we confirmed a differential expression tendency seen in RNAseq by using qRT-PCR with three biological replicates (Additional file 5: Figure S5A-D). Indeed, all genes that were shown to be down-regulated in RNAseq are also found to be down-regulated in qRT-PCR (around a 2-fold decrease at 10 h compared to 0 h). In contrast, all genes that were shown to be upregulated in RNAseq have their expression increased through time in qRT-PCR with comparable fold changes between the two methods.

## Discussion

Large-scale gene expression regulation during yeast-to-hypha (Y-to-H) transition in dimorphic fungi has been studied in model fungal species, such as *Saccharomyces cerevisiae* and *Candida albicans*, using RNAseq, DNA microarrays or phenotypic screening of mutants [33, 45, 55, 56]. It also has been recently investigated in *Penicillium marneffei* [42]*.* In *Ophiostoma novo-ulmi*, large-scale transcriptomic analyses have been performed to characterize molecular signatures of yeast and mycelial forms via Expressed Sequence Tags analyses [57] or, more recently, by RNAseq [48]. Here, we present the first whole-transcriptome study of a time-course experiment in which we monitored the Y-to-H transition in *O. novo-ulmi* using Ion Torrent RNAseq technology.

### Microscopy and flow cytometry

We first characterized the morphological switch process using various quantitative and qualitative techniques (light microscopy, scanning-electron microscopy, and flow cytometry) to assess the relevance of growth conditions that had been selected for the time-course experiment. Results show a greater degree of evidence for Y-to-H transition under the conditions that we have tested. We confirm the emergence of hyphae in culture by showing increasing cell size and the modification of single cell shapes in accordance with previous reports [9]. These changes are seen already within the first 4 h after transfer in a medium that induces Y-to-H. These results could be explained by the phenomenon of cell swelling at 2 h after transfer to static liquid OCM, prior to germination, which has been described earlier [9].

### Global view of transcriptomes of yeast-to-hypha transition

Second, in order to characterize transcriptome modifications during this major morphological change, we used mRNA sequencing. Interestingly, we observed large changes in gene expression among samples that were taken at different times during the Y-to-H transition, with almost 22% of *O. novo-ulmi* total gene content being differentially expressed. In particular, we found large transcriptomic variation between samples that were collected at the beginning of the experiment and those that were collected 2 h after transfer to static liquid OCM. While this difference agrees with the cell swelling and reshaping observed through microscopy and flow cytometry, it may also be related to the perception of the signal triggering the Y-to-H switch and the regulation of many processes that are required to achieve the transition. In contrast, between 10 and 27 h after transfer to static liquid OCM, molecular variability is relatively small, which could be explained by the fact that the percentage of hyphae that are produced is not increasing. Only the hyphae that were already present in the medium are growing between 10 and 27 h.

By analyzing processes that were enriched in the set of up-regulated genes during the morphological switch, we highlight biological processes that were related to major cell modification and remodeling. For instance, the activation of the cell cycle has already been shown in *S. cerevisiae* and *C. albicans* as being critical during morphological changes [58–60]. Furthermore, CAZyme genes are more likely to be actively transcribed than repressed, suggesting an active process of modifications in carbohydrate compounds, which is consistent with cell wall remodeling. By more in depth exploration of genes that are over-expressed during the morphological switch, we found genes that were related to specific hyphal structures, supporting the formation of hyphae. One of them is a gene that is predicted to encode a major protein (Hex1) that is associated with the formation of Woronin bodies. These organelles are associated with septal pores that are present in filamentous fungi [61, 62]. The *Hex1* gene appears to be conserved across species and has been shown to be over-expressed in hyphae of *Coccidioides* spp.[63]. In *Neurospora crassa*, Woronin bodies have been reported as being responsible for protecting hyphae against cellular damage [64, 65]. Even though their role has been clearly associated with stress response, Woronin bodies are found associated with septal pores but also in the cytoplasm of non-damaged hyphae in *A. fumigatus* [66]. Finally, in *Magnaporthe grisea*, the Hex1 protein is apparently required for efficient pathogenesis and in responses to nitrogen starvation, since partial deletion induced a reduction of the virulence and made the fungus unable to survive nitrogen starvation [67]. The *Hex1* gene is included in the Pathogen-Host interaction database (PHI-Base, www.phi-base.org) [68]. The high expression of *Hex1* in *O. novo-ulmi* at 27 h after induction of the transition suggests that the expression of this gene is correlated with hyphal formation. Putative actin-, tubulin- and septin-coding genes are also over-expressed through time, consistent with major cellular reorganization due to the formation of pluricellular structures and septa. Septins, in particular, have been reported to play an important role in the filamentous growth of other dimorphic fungi such as *Aspergillus nidulans*, *C. albicans* and *U. maydis* [69–72].

Interestingly, the most highly over-expressed gene, Onu3773, is related to the gene coding for the adhesin protein Mad1. In *Metarhizium robertsii* (previously *M. anisopliae* [73]), which is a well-known insect pathogen that can also colonize plant roots, Mad1 is involved in the adhesion of blastospores to the insect cuticle [74]. Also, the *Mad1* gene is highly expressed in the insect hemolymph [75]. It is also responsible for cytoskeleton organization, cell division and actin polymerization. All of these processes are over-represented in putative functions of genes that are over-expressed during the Y-to-H transition in *O. novo-ulmi*. Deletion of *Mad1* (Δ*Mad1*) in *M. robertsii* induces a delay in conidial germination, a reduction in blastospore formation, and the production of large multicellular hyphal bodies. Moreover, whereas *Mad1* is not expressed in freshly collected conidia, its expression increases as soon as conidia start swelling prior to germination [74]. Accordingly, in *O. novo-ulmi*, Onu3773 is not expressed 2 h after incubation under conditions promoting Y-to-H transition. However, after 4 h of incubation in static OCM, its expression level increases rapidly. This response could be connected with the cell swelling that was observed earlier in this study. Finally, *M. robertsii* Δ*Mad1* mutants are less virulent towards insects than the wild-type strain [74]. If the functions of the *Mad1* homolog in *O. novo-ulmi* are conserved, this gene could be a candidate for the link between morphology and virulence, although *O. novo-ulmi* itself is not entomopathogenic.

In contrast, gene Onu4296, which encodes Cerato-ulmin (CU), is down-regulated under our experimental conditions. Cerato-ulmin is a hydrophobin [76] that has been studied extensively in the context of DED [77–80]. Its role as a pathogenicity factor remains controversial, but it has been suggested that like Mad1 in *M. robertsii*, CU promotes the adherence of infectious spores to the cuticle of elm bark beetles [79]. The *CU* expression level was previously reported to be 20–60% higher in mycelium than in yeast cells that were grown in vitro [81]. Yet the CU gene was equally expressed in RNAseq data of yeast and mycelial forms [48]. Taken together, these contrasting results may reflect medium-dependent regulation and show the lack of conservation of expression for the *CU* gene among different studies, which in turn explains the controversy regarding the specific role of CU in pathogenicity.

Even though growth conditions and sequencing technologies differ from those that we used to compare yeast and mycelium growth phases [48], we found convincing overlaps between the two studies. For instance, genes such as Onu4877, Onu5171, Onu6217 and Onu7070, which are already described as being over-expressed in yeast or mycelium growth phases, show the same expression pattern and are over- or down-regulated during the Y-to-H transition. In total, we found 105 genes for which the regulation of gene expression appears to be conserved under variable growth conditions.

### Identification and expression analysis of *S. cerevisiae* MAPK, PKA, and RIM pathway homologs

We had previously shown that transcriptomes of yeast and mycelium phases of *O. novo-ulmi* are distinct from those of *H. capsulatum* and *C. albicans* [48]. However, we also found *O. novo-ulmi* homologs of genes encoding proteins involved in MAPK, PKA and RIM pathways. The role of the MAPK cascade in the Y-to-H transition has been studied extensively in model dimorphic fungal species such as *S. cerevisiae* [30, 82] (Fig. 1). We show that 11 key proteins have at least one homolog in *O. novo-ulmi,* of which four have two putative homologs (Ste20, Kss1, Sho1 and Msn4)*.* More interestingly, the expression of eight genes encoding these proteins is differentially regulated during the Y-to-H transition in *O. novo-ulmi.* Homologs of *Ste12*, *Kss1*, *Ste7* and *Ste20* are all over-expressed through time, consistent with data that were collected for *S. cerevisiae* [22]. Ste12 is a transcriptional factor that regulates the expression of multiple genes that are related to the formation of pseudohyphae in *S. cerevisiae* [83]. The *Ste12* homolog in *Candida albicans* is also involved in hyphal formation and its knockout mutation suppresses filamentation and virulence [39, 84]. During activation of pseudohyphal formation in *S. cerevisiae*, Ste12 acts as a heterodimer when combined together with Tec1 protein [85]. Here, the *Tec1* homologous gene (Onu5199) is not differentially expressed during the Y-to-H transition. This result might suggest that Tec1 is not required in the filamentation process in *O. novo-ulmi*. In contrast, upregulation of the *Ste12* homolog in *O. novo-ulmi* is consistent with a conserved role in the activation of hypha formation.

Moreover, the *Hog1* homolog is also over-expressed during Y-to-H transition in *O. novo-ulmi*. The MAPK encoded by this gene is described as a key regulator of hyper-osmolarity stress perception and is also related to morphological changes and virulence in *C. albicans* [34]. In *S. cerevisiae*, the Hog1 protein regulates invasive growth and contributes to the repression of pseudohyphal growth [33, 86]. Protein Msn4 of *S. cerevisiae* is a stress-responsive transcriptional activator activated by Hog1. This protein has two putative homologs in *O. novo-ulmi* (Onu8210 and Onu0070)*.* Interestingly, a paralog of *Msn4*, known as *Msn2*, is found in the genome of *S. cerevisiae* [87]. These two paralogs are largely functionally redundant and regulate stress response [88]. In *S. cerevisiae*, *Msn4* expression is induced by stress, whereas *Msn2* expression is constitutive [89]. Under our experimental conditions, the two homologs of *Msn4* have contrasting gene expression regulation. Gene Onu8210 is downregulated, whereas Onu0070 is upregulated. The latter is consistent with the upregulation of *Hog1* and of the entire MAPK cascade.

Gene Onu8210 encodes a putative transcriptional factor containing a zinc-finger DNA binding domain. Interestingly, this domain is not only homologous with the Msn4 domain but also with those from *S. cerevisiae* Mig1 and Nrg1 proteins. Both Mig1 and Nrg1 are negative regulators of genes that are involved in multiple processes, including filamentous growth of *S. cerevisiae* under glucose-limiting conditions [90, 91]. The downregulation of Onu8210 is consistent with the repression of a negative regulator of filamentatous growth, suggesting that this gene might encode a protein that acts more like Mig1 or Nig1 than Msn4.

Taken together, these results constitute the first report revealing potential involvement of the MAPK cascade in the process of morphological change in *O. novo-ulmi*. Further, they suggest a conserved mechanism across fungal families. Roles and activities for each of the proteins that are encoded by the identified genes must be further confirmed by functional analyses, such as gene knockout experiments, for which efficient protocols are still lacking in *O. novo-ulmi.*

Under the growth conditions that were selected for Y-to-H induction in *O. novo-ulmi*, gene Onu0836-*Yak1* is the only gene-encoding homolog of proteins involved in the PKA pathway in *S. cerevisiae* which is differentially expressed. Both genes encoding homologs of Phd1/Sok2 (Onu0977) and Tkp1-3 (Onu7708) showed variation in the number of reads between 0 and 4 h. Phd1 and Sok2 are actually paralogous proteins in *S. cerevisiae*, which explains why the homologous protein in *O. novo-ulmi* is the same for both proteins. The fact that all three putative catalytic sub-units of the PKA (Tpk1-3) have a unique homolog in *O. novo-ulmi* suggests that there is only one catalytic sub-unit in this species. In previous studies [13], cAMP was implicated in the dimorphism of *O. ulmi* NRRL6404. Over-expression of the gene encoding the downstream transcription factor of the PKA pathway, *Phd1* (*S. cerevisiae*) or *Efg1* (*C. albicans*), was sufficient to enhance filamentous growth in both species [22, 39, 92]. The slight modification in the expression of *Phd1* and *Tpk* homologs in *O. novo-ulmi* could reveal either no obvious role for these genes in the Y-to-H transition or that a very small increase in gene expression is sufficient for activation of the positive functions of both genes in the process of Y-to-H transition. In the latter case, the modification of *Tpk* expression might induce *Yak1* expression, which then positively regulates *Phd1* expression. The absence of a *Flo11* homolog in *O. novo-ulmi* suggests that there likely is an as yet unknown gene that is targeted by Phd1. Further studies monitoring cAMP concentrations during the Y-to-H transition would aid in demonstrating whether there truly is a relationship between gene expression and cAMP levels.

Finally, analyses of the six putative candidates for the RIM pathway in *O. novo-ulmi* revealed overexpression of the gene encoding the homolog of RIM101, which is the downstream transcriptional factor regulated by the RIM pathway in *S. cerevisiae* and *C. albicans* [28, 40]. We did not monitor changes in pH of the growth medium over the entire experiment. However, we noted that the pH of the 3-day-old pre-culture of yeast cells in OMM was 3.0, whereas fresh OCM that was used to induce transition to mycelium had a pH of 5.5 (data not shown). Therefore, modification in the expression of Onu0751-*RIM101* could be the result of the change in pH that was induced by the transfer to OCM, even though this modification is not as drastic as a switch from an acidic to a basic pH. Nevertheless, this also suggests that this gene is involved in the switch and, thus, has a conserved role among dimorphic fungal species.

## Conclusions

Our survey of morphological and transcriptomic changes during the yeast to hypha transition in *Ophiostoma novo-ulmi* highlights modifications in both the structure of cells and the regulation of gene expression within 10 h after induction of the switch. Analyses of gene expression regulation in a time-course experiment provided the first insights into the processes that were activated and those that were repressed during the Y-to-H transition in *O. novo-ulmi.* These analyses further confirmed the occurrence of major rearrangement of the cell wall and cellular structures. We pointed out both genes and pathways that are either specific to *O. novo-ulmi* or not described in model dimorphic fungi, (*Hex1*, *Mad1* or *CU*), or which converge in gene expression regulation for genes encoding proteins that are homologous to *Saccharomyces cerevisiae* proteins involved in the MAPK cascade related to the yeast to pseudohypha switch. Our experimental conditions also suggest the involvement of PKA and RIM pathways, which should be confirmed in future studies. Taken together, our results indicate many genes of interest for further investigations of the Y-to-H transition in *O. novo-ulmi* and highlight the possible convergence of molecular regulation of dimorphism between very divergent fungal species.

## Materials and methods

### Fungal growth conditions and sample collection

#### Preparation of RNAseq samples

Yeast cells (1 × 10^6^ spores mL^−1^) of *Ophiostoma novo-ulmi* ssp. *novo-ulmi* strain H327 (Centre d’Etude de la Forêt, fungal collection, http://www.cef-cfr.ca/index.php?n=CEF.Collections ChampignonsPathogenes) were first grown in liquid minimum medium (OMM) [93] that was supplemented with 1.15 g L^−1^ proline as the sole nitrogen source on an orbital shaker (Infors HT Ecotron) at 150 rpm (22 °C). After 3 days, cultures were filtered through 16 layers of sterile cheesecloth and diluted to 1 × 10^6^ spores mL^−1^ in flasks containing 100 mL of complete medium (OCM) with 1.15 g L^−1^ proline. Flasks were incubated under still conditions in darkness at room temperature (22 °C). At each point during the time-course experiment (0, 2, 4, 6, 10 and 27 h after transfer of cells to static liquid OCM), five photographs per flask were taken with a microscope (Olympus BX41, 400 × magnification) to measure the proportion of yeasts and hyphae. Cells were considered hyphal structures when they were at least twice as long as a typical yeast cell. About 60 structures were counted and measured at each time. One hundred milliliters of culture from each flask were transferred each time into two sterile Falcon 50 mL conical centrifuge tubes and centrifuged for 5 min at 4 °C and 3000 g. Pellets were resuspended in sterile water and transferred to 1.5 mL sterile microtubes. Samples were again centrifuged for 5 min at 4 °C and 3000 g, after which the pellets were flash-frozen in liquid N_2_. Samples were stored at −80 °C until RNA extraction. Three time series replicates were collected (18 samples in total).

#### Flow cytometry

Time points for flow cytometry measurements were 0, 2, 4, 6 and 10 h after transfer of cells to static liquid OCM. Samples that were incubated for 27 h in OCM were not analyzed since cell size and shape were no longer compatible with the flow cytometer. Cells were grown in OCM under the same conditions as for the RNAseq samples. At each time tested, cultures were diluted with sterile water in order to have 50–500 cells per μL in 300 μL of solution that was transferred to 96-well plates for analysis in a Guava® easyCyte HT Sampling flow cytometer (EMD Millipore Corp.). OMM that was supplemented with 1.15 g L^−1^ proline was used as negative control for the transition [7, 8].

#### Scanning electron microscopy (SEM)

Cells were grown and collected following the same procedure as for RNA extractions. Pellets of cells were resuspended in fixation buffer (2.5% glutaraldehyde in 0.1 M sodium cacodylate buffer, pH 7.3). Samples were fixed for 20 min at room temperature and stored at 4 °C for at least 12 h. Further processing of samples was performed at the Plateforme de Microscopie (IBIS/Université Laval) following the same procedure as described previously [94]. Samples were examined with a JEOL JSM6360LV microscope (JEOL Inc., Tokyo, Japan).

### RNA extraction, cDNA library production and RNA sequencing

Total RNA from each of the 18 samples was extracted using the Ambion® Ribopure™ Yeast kit (Life Technologies) (for schematic see Additional file 6: Figure S1). The quality was verified using a spectrophotometer (NanoDrop ND-1000, Thermo Scientific) and a bioanalyser (BioAnalyzer RNA 6000 n Kit, Agilent Technologies).

Complementary DNA (cDNA) libraries were constructed at the Plateforme d’Analyses Génomiques (IBIS/Université Laval) using the kapa stranded RNA-seq kit (Kapa Biosystems) with Y-adaptors that were custom made to be compatible with Ion Proton™ (Thermo Fisher Scientific) sequencing. Single-end RNA sequencing was performed following the manufacturer’s recommendations.

### RNAseq data preprocessing

RNAseq read quality was verified using FastQC software (http://www.bioinformatics.babraham.ac.uk/projects/fastqc/). Given the poor quality of 5’ and 3’ tails, 15 nt were removed in 5’ and sequences were trimmed to a length of 150 nt using Prinseq v0.20.4 [95]. Finally, sequences shorter than 25 nt were removed.

### RNAseq data analysis

Filtered reads were mapped onto the exon sequences of the *O. novo-ulmi* H327 genome [46] with TopHat2 (v.2.0.10) using default parameters [96]. For greater readability in the manuscript, genes of *O. novo-ulmi* are named “Onu” followed with the number of the gene. All further analyses were performed in the R statistical environment (v3.0.1; [97]). Raw data were filtered and transformed with the *Bioconductor* package *EdgeR* (correction by the library size and filtering of genes for which the row sum was less than 3 counts per million [CPM]) [98]. A multidimensional scaling (MDS) plot was used for the visualization of the variability among samples (function implemented in *EdgeR*), following the procedure described previously [48]. The MDS plot was produced using the R package *ggplot2* [99]. The updated version of *maSigPro* R package [50], *Next-maSigPro* [49], was used for the detection of differentially expressed genes (DEGs) in R. *P*-values were corrected using the False Discovery Rate (FDR) [51] with a threshold of 0.05.

Clustering analysis was performed using Short Time-series Expression Miner (STEM) software [52] on the set of DEGs with our own annotation file. The software assigns genes to a set of model profiles predefined in order to capture all the potential distinct patterns that can be expected from the experiment [53]. We chose a maximum number of 50 for model profiles with a significance threshold of 0.01 and False Rate Discovery (FDR) correction. Determination of differentially expressed genes was based on ‘Maximum − Minimum’ that is the largest difference in the gene’s value between any two time points, which are not necessarily consecutive [52]. A minimum fold change of two was required over time between normalized count values for a gene to be retained. Differential expression for each gene was calculated on the normalized count values, as:

$$ \mathrm{Differential}\ \mathrm{expression} = v(i)/v(0) $$

where v(i) is the average number of normalized counts at a time point of interest, and v(0) is the average number of normalized counts at the beginning of the experiment. Values less than 0 were inverted to ease of interpretation; in this case, the equation was written as:

$$ \mathrm{Differential}\ \mathrm{expression} = -1/\left(v(i)/v(0)\right) $$

Gene ontology (GO) term enrichment analyses were performed as described in Nigg and collaborators (2015) using the *Goseq* package in *Bioconductor 3.0* [100] and GO Trimming [101]. REVIGO was used to reduce redundancy and produce treemaps (space-filling visualization of hierarchical structures) using the *treemap* package in R [54]. Default parameters were used and the redundancy threshold (allowed similarity) was set at 0.4 (very small).

### Protein homology and gene orthology assessment

Sequences of MAPK, PKA and RIM proteins from *S. cerevisiae* were downloaded from NCBI and the yeast genome database (www.yeastgenome.com). A standalone version of *BLASTp* [102] was used (parameters: word-size = 3; e-value = 0.01) to assess homology with *O. novo-ulmi* proteins.

Orthologous genes among the identified homologs were highlighted by reciprocal best blast hits (RBH) using tblastx [102]. Exon sequences of the two species (*O. novo-ulmi* and *S. cerevisiae*) were compared using local databases and the RBH was conducted using the following parameters: e-value = 0.001 and word size = 5.

### Reverse transcription, PCR and quantitative Reverse Transcription-PCR (qRT-PCR)

Samples for qRT-PCR were collected following the procedure that was described above. Total RNA was extracted and samples were conserved at −20 °C for a maximum of 1 month prior to complementary DNA (cDNA) synthesis.

The cDNAs for qRT-PCR were synthesized by reverse transcription using Superscript II enzyme (Invitrogen) and according to the following protocol: 500 ng of mRNA (final volume of 8 μL, completed with nuclease-free water) were mixed with 1 μL of dNTPs 10 mM and 1 μL of oligo-dT 0.5 μg/μL. The mixture was incubated at 65 °C for 5 min. Samples were then cooled on ice for 1 min and quickly centrifuged. A mixture of 10X Buffer RT (2 μL), MgCl2 (25 mM, 4 μL), DTT (0.1 M, 2 μL), RNase Out (1 μL, Invitrogen) and Superscript II (200 U, 0.25 μL) was added to each sample. Preparations were gently mixed, quickly centrifuged and incubated for 50 min at 42 °C. Reactions were completed by incubating samples for 15 min at 72 °C. Samples were finally cooled down on ice for 5 min and 80 μL of nuclease-free water were added. cDNA samples were stored at −20 °C.

For each gene that was tested in qRT-PCR, primers for fragments that were 120–200 base pairs in length were designed using Primer3plus (http://primer3plus.com/cgi-bin/dev/primer3plus.cgi) and properties were verified with Oligocalc (http://www.basic.northwestern.edu/biotools/oligocalc.html) (Additional file 6: Table S1). Primers were then synthesized at the *Plateforme de séquençage et de génotypage des génomes* (Research Centre, Centre Hospitalier de l’Université Laval, Quebec QC, CA).

Prior to qRT-PCR, primers were tested on cDNA (9 ng μL^−1^) in PCR. Reaction volume of each sample was 25 μL (12.2 μL Premix Ex Taq TaKaRa, 1.25 μL of each primer, 5 μL of sterile water and 5 μL of cDNA). PCR conditions for amplification were: 10 s at 98 °C followed by 30 cycles of 10 s at 98 °C, annealing for 30 s at Tm (usually 55 °C), amplification for 12 s at 72 °C and a final step of 5 min at 72 °C. PCR products were transferred to 2% (m/v) agarose gel for electrophoresis (migration at 130 V) followed by staining in ethidium bromide bath for 15 min. DNA fragments were visualized under UV light.

Quantitative RT-PCR (qRT-PCR) reactions were performed with the PerfeCta™ SYBR® Green FastMix™, Low ROX kit (Quanta BioSciences) following the manufacturer’s instructions, with 10 μL of PerfeCta™ SYBR® Green FastMix™, Low ROX mix, 2.5 μL of 5’ and 3’ 1 μM primers for each gene that was tested and 5 μL of 1 ng μL^−1^ cDNA (or water for negative control) for a final volume of 20 μL in MicroAmp® Fast Optical 96-well plates (Applied Biosystems™). Gene expression was quantified using the 7500 Fast Real-Time PCR system (Applied Biosystems™). Genes Onu3626 (Ubiquitin conjugating enzyme), Onu1683 (Ubiquitin conjugating enzyme E2 6) and Onu0623 (RING finger ubiquitin ligase) were defined as reference genes using the R script of *NormFinder* software [103]. For each of the nine genes that were tested (Onu1392, Onu7491, Onu8395, Onu1002, Onu8210, Onu0980, Onu6970.1, Onu0303 and Onu5171), three biological replicates with three technical replicates were used to quantify expression with a comparative method (2^-ΔΔCt^). The three reference genes were used to calculate the level of transcripts at each time point (2 h, 4 h, 6 h and 10 h) relative to 0 h (beginning of the Y-to-H transition experiment).

## Additional files

Additional file 1: Figure S2. Distribution of the number of reads (log_10_ scale) per gene, per condition (mean of 3 replicates). (PNG 76 kb)
Additional file 2: Tables S1-S7.
**Table S1**: qRT-PCR primers for reference genes and tested genes. **Table S2**: Results of BLASTp analysis of Saccharomyces cerevisiae dimorphism related proteins with Ophiostoma novo-ulmi genome. The last column indicates if the homologous proteins are orthologs (results of reciprocal best blast hits (RBH) using tblastx on exon sequences for each gene of the two species, e-value = 1x10^3 and word size = 5). **Table S3**: Normalized read counts for the 7605 genes analyzed using EdgeR and maSigPro in R. 1-3: 0 h; 4-6: 2 h; 7-9: 4 h; 10-12: 6 h; 13-15: 10 h; 16-18: 27 h. DEGs : Differentially Expressed Genes detected using maSigPro, FDR ≤ 0.05. For each DEG, the number of the STEM profile associated is indicated. Nigg et al. 2015: genes overexpressed in Yeast or Mycelium growth phases in the study of Nigg and collaborators in 2015 [47]. **Table S4**: Description of the 815 genes found in the STEM profile 39. Values are means of differential gene expressions for each time compared to 0 h.**Table S5**: Enriched biological processes in the STEM profile 39 containing at least 4 DEGs genes after filtration using GO Trimming and REVIGO. GO term in bold are parent term retained as name for a regroupment. **Table S6**: Description of the 256 genes found in the STEM profile 8. Values are means of differential gene expressions for each time compared to 0 h. **Table S7**: Enriched biological processes in the STEM profile 8 containing at least 4 DEGs genes after filtration using GO Trimming and REVIGO. GO term in bold are parent term retained as name for a regroupment. (XLSX 1029 kb)
Additional file 3: Figure S3. REVIGO Gene Ontology treemap for the 112 biological processes that were enriched in profile 39. The size of boxes represents the absolute *P*-value for enrichment of each GO term in the gene set of the profile (log_10_). (PNG 39 kb)
Additional file 4: Figure S4. REVIGO Gene Ontology treemap for the 22 biological processes enriched in profile 8. The size of boxes represents the absolute *P*-value for enrichment of each GO term in the gene set of the profile (log_10_). (PNG 27 kb)
Additional file 5: Figure S5. Quantitative Reverse Transcriptase-Polymerase Chain Reaction (qRT-PCR) results at 0, 2, 4, 6, and 10 h following transfer to fresh complete growth medium (OCM) for A, downregulated genes; B, C and D, over-expressed genes. Differential expression: 2^-ΔΔCt^, fold increase in expression level compared to the start of the experiment (0 h). Standard deviations are calculated on three biological replicates for each gene. Three technical replicates per gene were run. Transcript levels were normalized with three control genes (Onu3626, Onu1683, Onu0623) that had the most stable expression and were used to calculate a reliable normalization factor according to *Normfinder* software [103]. (PNG 161 kb)
Additional file 6: Figure S1. Workflow for RNAseq data production and analysis. (PNG 115 kb)
