# Supplementary figures and images for: From yeast to hypha: defining transcriptomic signatures of the morphological switch in the dimorphic fungal pathogen Ophiostoma novo-ulmi
Source: BMC Genomics. 2016 Nov 15;17:920. doi: 10.1186/s12864-016-3251-8 (PMC5111228; doi:10.1186/s12864-016-3251-8)

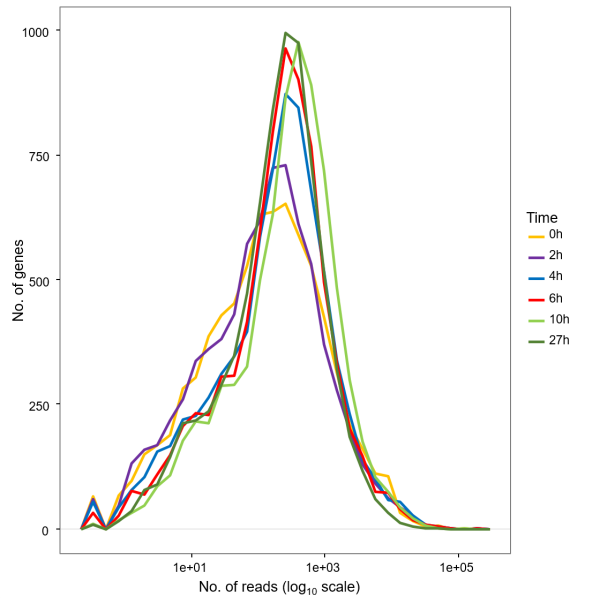

Supplement: Additional file 1: Figure S2. — Distribution of the number of reads (log10 scale) per gene, per condition (mean of 3 replicates). (PNG 76 kb) [file 12864_2016_3251_MOESM1_ESM.png]

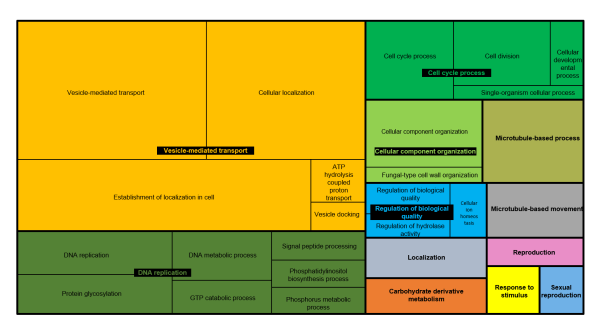

Supplement: Additional file 3: Figure S3. — REVIGO Gene Ontology treemap for the 112 biological processes that were enriched in profile 39. The size of boxes represents the absolute P-value for enrichment of each GO term in the gene set of the profile (log10). (PNG 39 kb) [file 12864_2016_3251_MOESM3_ESM.png]

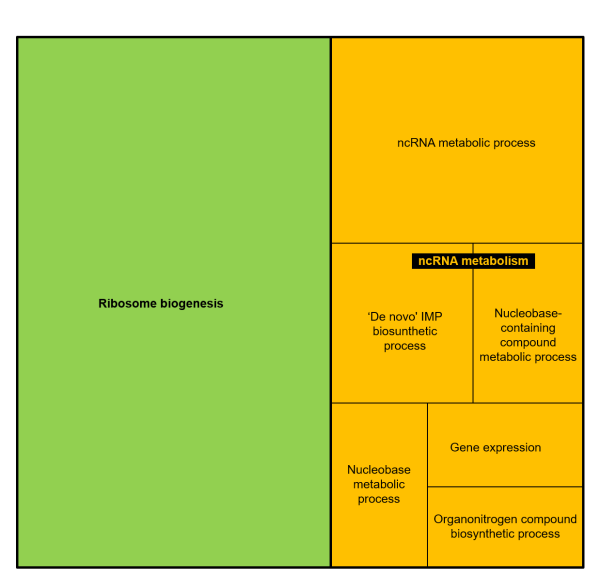

Supplement: Additional file 4: Figure S4. — REVIGO Gene Ontology treemap for the 22 biological processes enriched in profile 8. The size of boxes represents the absolute P-value for enrichment of each GO term in the gene set of the profile (log10). (PNG 27 kb) [file 12864_2016_3251_MOESM4_ESM.png]

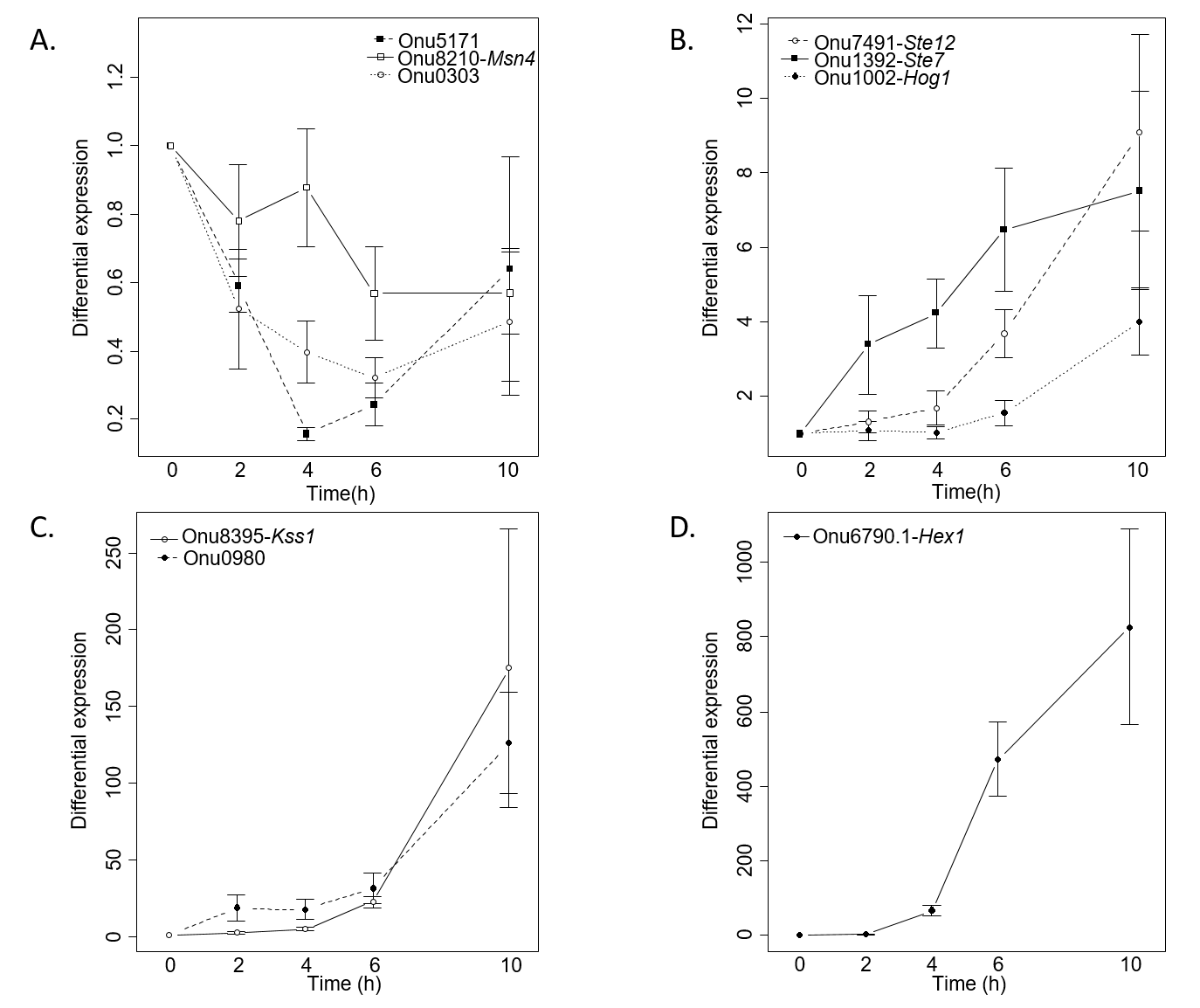

Supplement: Additional file 5: Figure S5. — Quantitative Reverse Transcriptase-Polymerase Chain Reaction (qRT-PCR) results at 0, 2, 4, 6, and 10 h following transfer to fresh complete growth medium (OCM) for A, downregulated genes; B, C and D, over-expressed genes. Differential expression: 2-ΔΔCt, fold increase in expression level compared to the start of the experiment (0 h). Standard deviations are calculated on three biological replicates for each gene. Three technical replicates per gene were run. Transcript levels were normalized with three control genes (Onu3626, Onu1683, Onu0623) that had the most stable expression and were used to calculate a reliable normalization factor according to Normfinder software [103]. (PNG 161 kb) [file 12864_2016_3251_MOESM5_ESM.png]

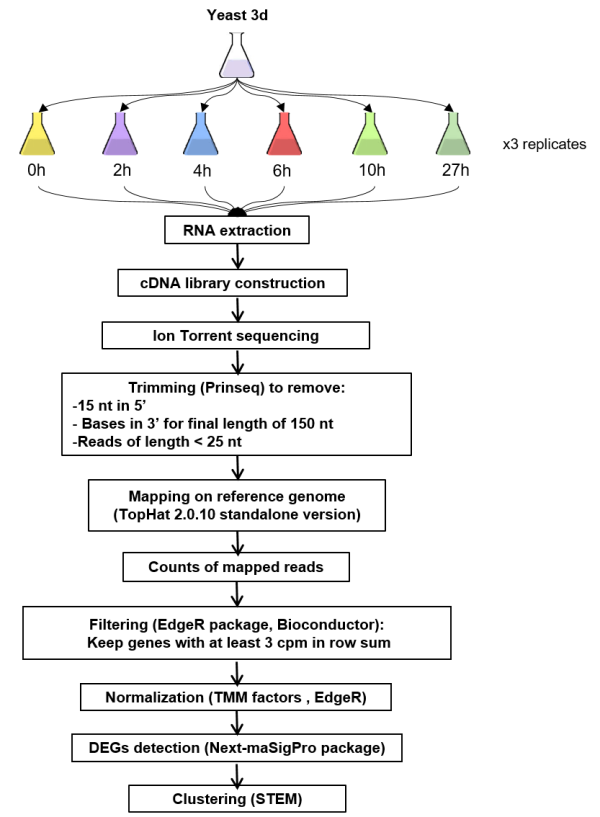

Supplement: Additional file 6: Figure S1. — Workflow for RNAseq data production and analysis. (PNG 115 kb) [file 12864_2016_3251_MOESM6_ESM.png]
